# An Effective Electrochemical Platform for Chloramphenicol Detection Based on Carbon-Doped Boron Nitride Nanosheets

**DOI:** 10.3390/bios13010116

**Published:** 2023-01-09

**Authors:** Jingli Yin, Huiying Ouyang, Weifeng Li, Yumei Long

**Affiliations:** 1 College of Chemistry, Chemical Engineering and Materials Science, Soochow University, Suzhou 215123, China; 2 The Key Lab of Health Chemistry and Molecular Diagnosis of Suzhou, Soochow University, Suzhou 215123, China

**Keywords:** hexagonal boron nitride, carbon doping, chloramphenicol, differential pulse voltammetry, molten salt synthesis

## Abstract

Currently, accurate quantification of antibiotics is a prerequisite for health care and environmental governance. The present work demonstrated a novel and effective electrochemical strategy for chloramphenicol (CAP) detection using carbon-doped hexagonal boron nitride (C-BN) as the sensing medium. The C-BN nanosheets were synthesized by a molten-salt method and fully characterized using various techniques. The electrochemical performances of C-BN nanosheets were studied using cyclic voltammetry (CV) and electrochemical impedance spectroscopy (EIS). The results showed that the electrocatalytic activity of *h*-BN was significantly enhanced by carbon doping. Carbon doping can provide abundant active sites and improve electrical conductivity. Therefore, a C-BN-modified glassy carbon electrode (C-BN/GCE) was employed to determine CAP by differential pulse voltammetry (DPV). The sensor showed convincing analytical performance, such as a wide concentration range (0.1 µM–200 µM, 200 µM–700 µM) and low limit of detection (LOD, 0.035 µM). In addition, the proposed method had high selectivity and desired stability, and can be applied for CAP detection in actual samples. It is believed that defect-engineered *h*-BN nanomaterials possess a wide range of applications in electrochemical sensors.

## 1. Introduction

Chloramphenicol (CAP) is a nitrobenzene derivative and was first obtained from *Streptomyces venezuelae* in 1947 [1]. This compound is highly active against a variety of bacteria and thus has been widely employed as an antibiotic to treat various diseases [2,3]. However, the abuse of CAP has been a significant threat to environmental security and human health. For instance, CAP can easily accumulate to cause persistent environmental hazards due to its poor biodegradability [4,5]. Furthermore, these CAP residuals enter the human body through the food chain and result in serious diseases [6,7]. For this reason, the use of CAP in aquatic products and animal feeds has been prohibited in many countries [8]. Unfortunately, CAP residuals have still been detected in biological and environmental samples because of their low cost and high effectiveness [9,10,11]. Accordingly, it is imperative to develop an effective method for CAP detection.

Spectrophotometric and chromatographic methods have been developed for CAP detection [12,13,14,15,16,17]. Nevertheless, these methods are always time-consuming, expensive, and have cumbersome preprocessing [18]. Hence, most researchers have focused on electrochemical techniques due to their merits of simple preparation, fast response, good stability and in situ analysis [19,20]. Chloramphenicol is an electroactive substance and is able to produce an electrochemical signal when the redox reactions of CAP occur at the surface of an electrode. Generally, a bare GCE only gives rise to a poor electrochemical response toward CAP. To ensure high sensitivity and selectivity, chemical modification with appropriate electrocatalytic materials is necessary for GCEs. The surface modification is capable of accelerating charge migration and providing abundant active sites for further promoting electrochemical reactions. Consequently, tremendous efforts have been dedicated to preparing catalytic materials for the construction of electrochemical sensors. 

Over the past decades, two-dimensional (2D) materials have been extensively exploited for the fabrication of electrochemical sensors based on their fascinating physiochemical performances [21,22,23]. Hexagonal boron nitride (*h*-BN) has a typical 2D layered structure and this compound has found widespread applications due to its high thermal conductivity, good lubricity, outstanding mechanical strength, and chemical stability [24,25,26]. Different from graphite of a semi-metallic nature, *h*-BN has a wide bandgap (~5.9 eV) because its π electrons are localized [27,28]. The applications of *h*-BN in functional devices have been limited due to its poor electron transport capability. Interestingly, the electronic properties of BN strongly rely on its microstructure, such as dimensionality, bond configuration and defects [29,30,31]. Among them, engineering defects is an effective way to ameliorate the photoelectrochemical properties of *h*-BN because they can form defect levels in the forbidden band to alter the energy band structure [32,33]. Recently, we demonstrated that point defects induced by heterovalent ion doping have great potential to improve sensing properties in *h*-BN systems [34,35]. Theoretical calculations also suggested that carbon doping can functionalize *h*-BN and makes it potentially applicable in the catalyst and sensor fields [36,37,38]. To the best of our knowledge, no studies on *h*-BN nanomaterials for the electrochemical detection of CAP have been reported.

In this work, a novel CAP electrochemical sensor was fabricated using C-BN nanosheets as the sensing medium. The C-BN nanosheets were prepared by a molten salt method and systematically characterized. Carbon doping enlarged the electrochemically active surface area (EASA) and facilitated electron transfer, which are favorable to the reaction kinetics. Therefore, enhanced electrochemical properties of *h*-BN were achieved upon doping with carbon. C-BN/GCE was employed for CAP detection, and its analytical performance was studied in detail. We also demonstrated the practical applications of the developed electrochemical sensor for CAP detection in real samples.

## 2. Materials and Methods

### 2.1. Materials

Cyanuramide (C_3_N_6_H_6_), H_3_BO_3_, *h*-BN nanoparticles and chloramphenicol were obtained from Aladdin (Shanghai, China). Glucose, ZnCl_2_, CuCl_2_·2H_2_O, NaNO_3_, NaNO_2_, NaH_2_PO_4_, Na_2_HPO_4_, KCl, K_3_[Fe(CN)_6_], and K_4_[Fe(CN)_6_] were purchased from Sinopharm Chemical Reagent Co., Ltd. (Shanghai, China). Human serum, dopamine hydrochloride (DA), cysteine (Cys), chlortetracycline (CTC), and acetaminophen (AAP) were purchased from Shanghai Yuanye Bio-Technology Co., Ltd. Chloramphenicol eye drops (Runshu, Specification: 25 mg/10 mL) were produced by BAUSCH & LOMB Freda Pharmaceutical Co., Ltd. (Jinan, China). The Al_2_O_3_ polishing powder and glassy carbon electrodes (GCE, ϕ = 0.3 cm) were obtained from Shanghai Chenhua Co., Ltd. (Shanghai, China). All reagents were used as received. Deionized (DI) water was adopted for solution preparation throughout the work. Electrochemical tests were conducted in 0.2 M phosphate buffer saline (PBS), which were prepared by mixing an appropriate amount of NaH_2_PO_4_ (0.2 M) and Na_2_HPO_4_ (0.2 M).

### 2.2. Equipment

Powder X-ray diffraction (XRD) patterns were obtained on a D8 Bruker X-ray diffractometer with a Cu-K_α1_ radiation source. Morphology, particle size and EDS analysis were investigated with an FEI Tecnai G20 transmission electron microscope (TEM). Fourier transform infrared (FTIR) spectra were acquired from a Nicolet 550 spectrometer. An F-4500 fluorescence spectrometer was used to test the photoluminescence (PL) properties of the samples. The UV–Vis diffuse reflectance spectroscopy (DRS) was recorded with a UV3600 spectrophotometer (Shimadzu Corporation, Japan).

A CHI 660E workstation (Shanghai China) was utilized for electrochemical tests, which were performed in conventional three-electrode installation at room temperature (25 ± 2 °C). The electrochemical installation consists of a saturated calomel electrode (SCE, reference electrode) and a platinum wire (counter electrode). Unmodified/modified GCEs were adopted as working electrodes. The CV and EIS measurements were carried out in a KCl solution (0.1 M) containing 1 mM [Fe(CN)_6_]^3−/4−^. The DPV plots were acquired in PBS solution. The scanning parameters were −0.8–−0.4 V for potential window, 50 mV for modulation amplitude, 4 mV for potential increment and 0.05 s for pulse width.

### 2.3. Synthesis of Carbon-Doped BN

Carbon-doped boron nitride (C-BN) was prepared modified from a previous report [35]. In general, cyanuramide aqueous solution is weakly basic, and H_3_BO_3_ is weakly acidic. They can interact with each other through acid-base interactions to form C_3_N_6_H_6_·2H_3_BO_3_ [39]. To successfully introduce carbon sources, excessive cyanuramide was added to the reaction. In a typical procedure for preparing carbon-doped BN nanosheets, 3.0 g H_3_BO_3_ was dissolved into 200 mL DI water and heated to 95 °C (water bath). After that, 5.0 g cyanuramide was added into the hot boric acid solution partially three times. The above mixture was vigorously stirred (95 °C) for 2 h and then subjected to the removal of water by vacuum rotary evaporation. The solid mixture was dried overnight at 80 °C and crushed to obtain a white precursor. Afterwards, 2 g precursor was well-mixed with 10 g NaCl/KCl (mass ratio of 45/55) eutectic matrix and transferred to a high-alumina crucible. The mixture was calcined at 900 °C for 3 h under a reducing atmosphere (carbon). To remove free carbon residues, the powders were washed with hot dilute nitric acid (0.2 M) and hot water several times. The final yellow powder was ground and dried and named C-BN.

### 2.4. Construction of the C-BN/GCE Sensor

The C-BN/GCE was obtained using a casting method. First, successive mirror polishing of the GCE was carried out on a suede using Al_2_O_3_ slurries (300 and 50 nm) and cleaned with ethanol and deionized water. Next, the cleaned GCE was treated in 1.0 M H_2_SO_4_ solution until a stable cyclic voltammetry curve was obtained. Meanwhile, C-BN aqueous suspensions (1 mg/mL) were obtained by dispersing C-BN nanosheets in deionized water under ultrasonication. Then, the suspension was spread on the surface of the pretreated GCE and dried naturally. Finally, the C-BN/GCE was carefully rinsed with deionized water to remove any unbound compounds. As a reference, an *h*-BN-modified GCE (*h*-BN/GCE) was constructed with the same method.

### 2.5. Sample Preparation

A CAP stock solution (10 mM) was freshly obtained by dissolving 0.323 g CAP in 100 mL deionized water and stored at 4 °C in the dark. The practicability of the proposed method was investigated by analyzing CAP in real samples (chloramphenicol eye drops and human serum), which were first diluted using 0.2 M PBS (pH 7.0). The final CAP concentrations were then examined by the C-BN/GCE sensor using a standard addition method.

## 3. Results and Discussion

### 3.1. Characterization

**Figure 1:**
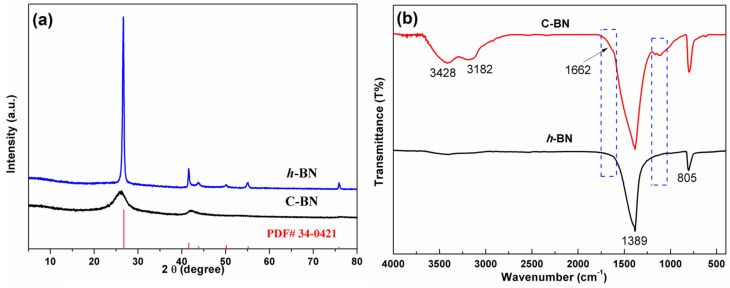
(**a**) XRD patterns and (**b**) FTIR spectra of *h*-BN and C-BN.

To identify the crystal structure of the products, powder XRD measurements were performed. Figure 1a displays the XRD patterns of C-BN and *h*-BN. As a reference, the standard diffraction data (JCPDS No. 34–0421) of hexagonal BN are also provided in Figure 1a. For both samples, all the diffraction peaks could be well indexed by standard diffraction data, and no additional diffraction peaks were found, indicating good phase purity. Different from *h*-BN, C-BN showed weak and broad diffraction peaks, suggesting that the hexagonal crystal structure was disturbed by carbon doping. A peak shift was also observed in the C-BN XRD pattern, which is attributed to the successful incorporation of carbon in the *h*-BN structure [39,40]. In the FTIR spectra (Figure 1b), both C-BN (red curve) and *h*-BN showed two strong absorption bands at 1389 and 805 cm^−1^, which should be ascribed to the in-plane B-N stretching and out-of-plane B-N-B bending vibrations [41,42]. For the C-BN sample, some new IR absorption bands were visible. Several bands in the 1000–1210 cm^−1^ range can be assigned to C-N and C-B vibrations, and C = N vibrations contribute to the absorption band at 1662 cm^−1^ [40,42]. The results suggested that carbon atoms were successfully incorporated into the *h*-BN crystal. On the other hand, the existence of O-H and N-H was evidenced by absorption bands at 3428 cm^−1^ and 3182 cm^−1^. These functional groups are beneficial for adsorbing target objects for further catalytic reactions.

**Figure 2:**
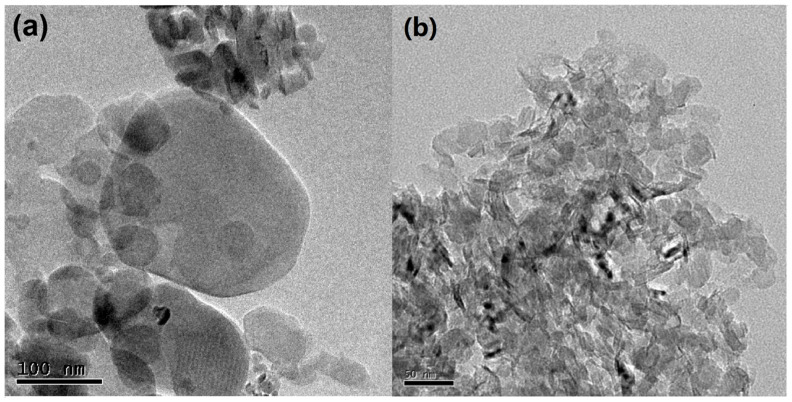
TEM images of (**a**) *h*-BN and (**b**) C-BN.

Figure 2 displays TEM images of the *h*-BN and C-BN samples. Both samples are composed of sheet-like materials. These nanosheets were highly transparent under the electron beam, implying their ultrathin features. Compared to that of *h*-BN (Figure 2a), C-BN (Figure 2b) exhibited a more uniform morphology and smaller size, which would provide a more active surface for adsorbing target compounds. Figure S1 shows the EDS profile of C-BN, confirming the presence of B, N and C.

**Figure 3:**
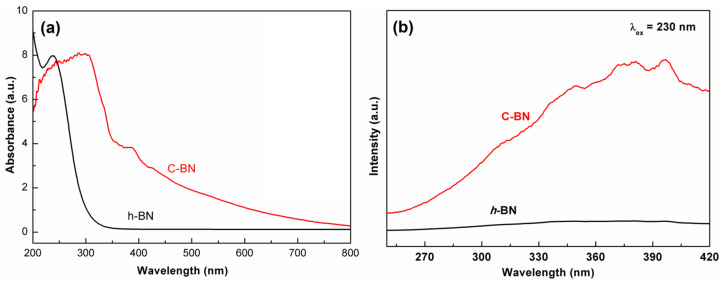
(**a**) UV–Vis DRS and (**b**) emission spectra (excited by 230 nm) of C-BN (red curve) and *h*-BN (black curve).

The band structure of semiconductors plays an important role in their electrocatalytic activity. The DRS was measured to investigate the influence of carbon doping on the band structure. As shown in Figure 3a, there was a drastic absorption in the ultraviolet window for both samples, which corresponds to the bandgap energy of a semiconductor. Compared to that of *h*-BN, the absorption edge of C-BN obviously shifts to the low energy direction. In addition, a wide absorption band appeared in the whole visible range for the C-BN sample. The results indicate that carbon doping has a significant impact on the band structure of *h*-BN and then modulates its photoelectronic properties. Figure 3b shows the PL spectra of the *h*-BN and C-BN samples under excitation at 230 nm. The C-BN shows multiple emission bands, which were not observed for *h*-BN. These emission peaks of C-BN were ascribed to carbon-linked defects, which modulated the behaviors of electrons [43].

### 3.2. CV and EIS Studies

**Figure 4:**
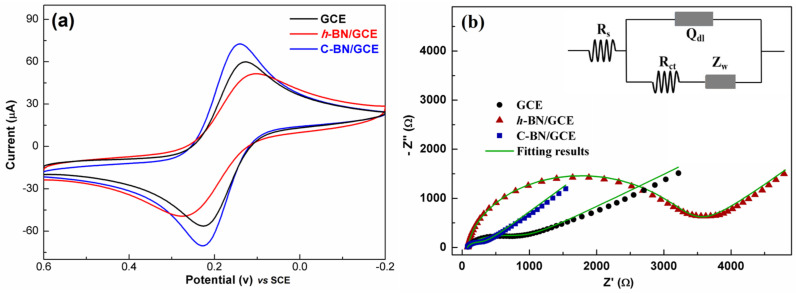
(**a**) CV and (**b**) EIS plots of 1 mM [Fe(CN)_6_]^3−/4−^ in 0.1 M KCl solution for GCE, *h*-BN/GCE and C-BN/GCE. Inset displaying the equivalent circuit for EIS tests.

Figure 4a shows the CV response of different electrodes. Each electrode demonstrated a pair of well-defined redox peaks, which resulted from the redox of [Fe(CN)_6_]^3−/4−^. Among all the electrodes, C-BN/GCE exhibited the sharpest and highest redox peaks. This reveals that the electron transfer rate of C-BN/GCE is faster than that of the others. In addition, the EASA of the electrode was calculated based on the dependence of the CV on the scan rate (Figure S2a,c,e) using the Randles–Sevcik equation [44]

*I*_p_ = 2.69 × 10^5^·(*n*^3/2^) *A*·*D*^1/2^·*C*·ν^1/2^ (1)

where *I*_p_ is the peak current and *ν* is the scan rate (V/s). For 1 mM [Fe(CN)_6_]^3−/4−^ (*C*) in 0.1 M KCl solution, *n* equals 1 and the diffusion coefficient (*D*) is 6.5 × 10^6^·cm^2^/s. Accordingly, the EASA (*A*) of electrodes can be estimated based on the fitting results between *I*_p_ and ν^1/2^ (Figure S2b,d,f). The EASA values of GCE, *h*-BN/GCE and C-BN/GCE were 0.19 cm^2^, 0.17 cm^2^, and 0.23 cm^2^, respectively. The good electron transfer ability and large EASA of C-BN/GCE should be ascribed to the defect structures induced by carbon-doping, as they not only modulate the electronic structure of *h*-BN but also provide more active sites for electrochemical reactions.

The EIS technique was utilized to study the interface nature of different electrodes, and the results are shown in Figure 4b. In the Nyquist plot, each curve consists of a semicircle part and a linear part. The semicircle diameter is proportional to the charge transfer resistance (R_ct_) of an electrode, reflecting the electron transport ability at the electrode/electrolyte interface. As shown in Figure 4b, there are significant differences between the plots, revealing the distinct electrochemical behaviors of these electrodes. By fitting with the equivalent Randle circuit (inset of Figure 4b), the R_ct_ values are 843 Ω, 3383 Ω and 275 Ω for the GCE, *h*-BN/GCE and C-BN/GCE, respectively. The R_ct_ of C-BN/GCE was less than that of the other electrodes. This result is consistent with the above CV data, indicating the outstanding charge transferability of C-BN/GCE. Therefore, it is expected that carbon-doped BN has potential applications in electrochemical sensors.

### 3.3. Electrochemical Behaviors of CAP over C-BN/GCE

**Figure 5:**
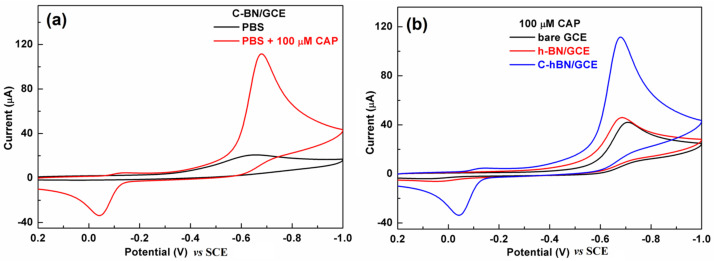
(**a**) CVs of C-BN/GCE in the absence/presence of 100 μM CAP; (**b**) CVs of GCE, *h*-BN/GCE and C-BN/GCE in the presence of 100 µM CAP. (Conditions: 0.2 M PBS with pH of 7.0 at a scan rate of 100 mV/s).

The electrochemical behaviors of CAP over different electrodes were first investigated by CV in 0.2 M PBS (pH = 7.0) with a scan rate of 100 mV/s. Figure 5a displays CV profiles obtained at the C-BN/GCE in the absence/presence of 100 µM CAP. No redox signals were discerned in the absence of CAP. In contrast, there was a pair of redox peaks in the presence of 100 µM CAP with an oxidation peak at −0.039 V and a reduction peak at −0.675 V (vs. SCE), respectively. The −0.675 V peak resulted from the nitro group reduction of CAP to hydroxylamine and the −0.039 V was ascribed to the oxidation of hydroxylamine to a nitroso group [45]. As depicted in Figure 5b, both the GCE and *h*-BN/GCE exhibited similar voltammetric signals toward CAP because they are electroactive. Interestingly, the redox signal produced by C-BN/GCE was remarkably larger than those obtained from bare GCE and *h*-BN/GCE. Its reduction peak current (88.9 µA) was approximately 2.6 times that of *h*-BN/GCE (34.1 µA) and 2.7 times that of GCE (32.9 µA). The favorable electrocatalytic effect of C-BN in the redox of CAP is attributed to its defect structure induced by carbon doping, which facilitates electron transfer and provides abundant active sites. Therefore, C-BN/GCE shows high sensitivity in the detection of CAP. As the reduction step of CAP at −0.68 V exhibited a better current response compared to the others, this peak was adopted for later experiments.

### 3.4. Effect of Scan Rate

**Figure 6:**
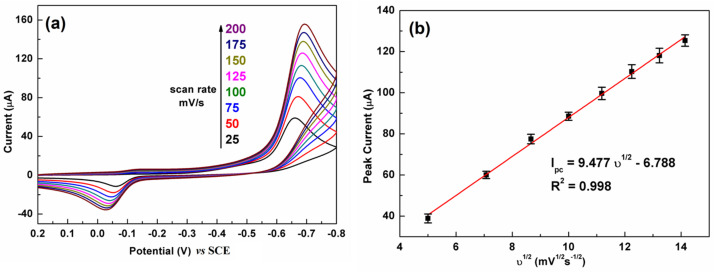
(**a**) The effect of scan rate on the CV response of the C-BN/GCE toward CAP (100 μM) and (**b**) corresponding linear plot of reduction currents against square root of scan rate. (Conditions: 0.2 M PBS with pH of 7.0).

Figure 6 illustrates the influence of the scan rate on the CV response of C-BN/GCE toward 100 µM CAP. The test conditions were 0.2 M PBS and pH 7.0. Figure 6a shows that the reduction peak current has a positive relation to the scan rate from 25 to 200 mV/s. A linear relationship between the reduction peak current and the square root of the scan rate (υ^1/2^) was observed (Figure 6b). The regression equation was defined as I_pc_ = 9.477 υ^1/2^ − 6.788. The good linear relationship (R^2^ = 0.998) revealed that electrochemical reduction of CAP over C-BN/GCE is a diffusion–controlled process [2].

### 3.5. Effect of pH

**Figure 7:**
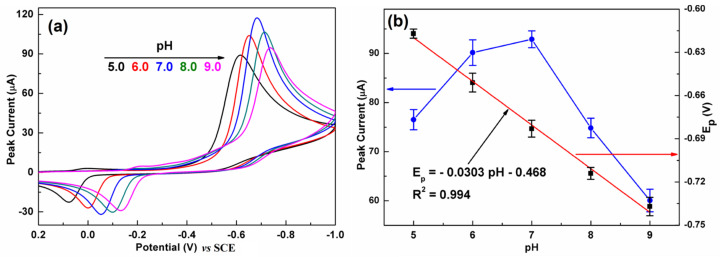
(**a**) CVs of C-BN/GCE at different pH (5.0, 6.0, 7.0, 8.0 and 9.0). (**b**) Dependence of the reduction peak current (left, blue curve) and E_pc_ (right, black dots) on pH, and corresponding fitting results (red plot). (Conditions: 0.2 M PBS containing 100 µM CAP at a scan rate of 100 mV/s).

It is well known that the pH of the electrolytes plays an important role in an electrochemical reaction. Figure 7a presents the influence of pH on the CV response of C-BN/GCE toward CAP. The current response gradually increased with increasing pH from 5.0 to 7.0 and then significantly decreased with further increasing pH (Figure 7b, left). Meanwhile, the larger pH value makes the reduction peak potential shift to a more negative direction, implying that protons participated in the electrochemical reaction. As illustrated in Figure 7b (right), the peak potential showed a good linear relationship to pH with a regression equation of I_pc_ = −0.0303 pH–0.468 (R^2^ = 0.994). The slope value of −0.0303 V/pH is very close to the theoretical value of −0.059/2 V/pH. Therefore, the electroredox of CAP over C-BN/GCE is an irreversible process involving one electron and two protons, which is consistent with previous reports [45,46]. According to literature [47], the pH value has an impact on the structure of CAP and further on its adsorption on the electrode surface. On the other hand, increasing the pH of the alkaline electrolyte could inhibit the reduction reaction of CAP. As a result, the best current response was achieved at pH 7.0, and was adopted for the later studies. The possible electrochemical reaction mechanism of CAP on C-BN/GCE can be described by Figure S3 [2].

### 3.6. Detection of CAP Using the DPV Method

**Figure 8:**
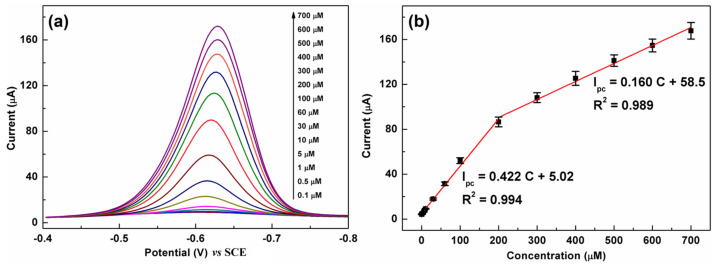
(**a**) DPV responses of C-BN/GCE toward different CAP concentrations; (**b**) corresponding linear plots of I_pc_ vs. CAP concentration (Circle dots are experimental data and red lines are fitting results). (Conditions: 0.2 M PBS with pH of 7.0, scanning window: −0.8–−0.4 V, modulation amplitude: 50 mV, potential increment: 4 mV, pulse width: 0.05 s).

The DPV technique is more effective and sensitive than CV for the quantification of organic substances and thus was employed for CAP detection. Figure 8a shows a series of DPV curves of C-BN/GCE for different CAP concentrations. The current response monotonically increased with increasing CAP concentration from 0.1 µM to 700 µM. Figure 8b illustrates the linear calibration plots between the peak current and concentration. The regression equations can be described as I_pc_ (µA) = 0.422 C (µM) + 5.02 (R^2^ = 0.994) in the low concentration region (0.1–200 µM) and I_pc_ (µA) = 0.160 C (µM) + 58.5 (R^2^ = 0.989) in the high concentration region (200–700 µM). The limit of detection (LOD) was calculated using the equation LOD = 3σ/S according to the IUPAC definition [18], in which σ is the standard deviation of 5 blank measurements and S is the slope of the calibration plot. The proposed method shows a low LOD of 0.035 µM. The analytical performance of C-BN/GCE was compared with that of other electrodes reported previously. As summarized in Table 1, C-BN/GCE is a suitable platform for CAP detection. Moreover, the construction procedure of C-BN/GCE is facile, and all elements, including carbon, boron, and nitrogen, are earth-abundant.

### 3.7. Selectivity and Stability

**Figure 9:**
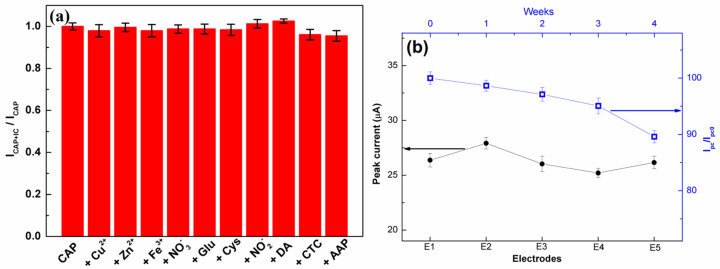
(**a**) Anti-interference ability of C-BN/GCE in the determination of CAP (50 µM CAP, 10-fold concentrations of Zn^2+^, Cu^2+^, Fe^3+^, NO_3_^−^, glucose, Cys, NO_2_^−^, DA, CTC, and AAP; (**b**) Left (black curve): Reproducibility of the proposed sensor; Right (blue curve): Evaluation of the long-term stability of the proposed sensor for four weeks (Circle and square symbols are experimental data). All tests were performed in 0.2 M PBS at pH 7.0 using the DPV method.

The selectivity of the C-BN/GCE for the electrocatalytic redox of CAP was investigated to ensure its practical applications. The DPV measurements were conducted using C-BN/GCE to detect 50 µM CAP in the absence/presence of various interfering compounds (IC), such as 10-fold concentrations of Zn^2+^, Cu^2+^, Fe^3+^, NO_3_^−^, glucose, Cys, NO_2_^−^, DA, CTC, and AAP. Figure 9a displays the ratios of peak current before and after adding various interferents. All the investigated compounds had a negligible influence on the current response of CAP with a current deviation of less than 5.0%, indicating the good selectivity of the developed method. The satisfied selectivity of the proposed method should be ascribed to the π-π interaction and specific surface adsorption of CAP molecules by C-BN nanosheets.

Five different C-BN/GCEs were fabricated independently through the same process and used for CAP detection under the same conditions. All the electrodes displayed similar peak current values with a relative standard deviation (RSD) of 2.76% (Figure 9b, left), confirming the favorable reproducibility of the proposed method. Meanwhile, ten cycle tests were performed on the same C-BN/GCE in the presence of 50 µM CAP. The RSD of the current responses was 2.44%. Therefore, the modified electrode possesses prominent repeatability. For long-term stability, the current response of the same C-BN/GCE toward 50 µM CAP was measured every week. After 4 weeks of storage at room temperature, the retention rate of the peak current was 89.6% (Figure 9b, right), demonstrating that the sensor was stable.

### 3.8. Real Sample Analysis

To assess the practical feasibility of the proposed method, C-BN/GCE was applied to detect CAP in real samples using a standard addition method. Real samples (human serum and eye drops) were first diluted using 0.2 M PBS (pH 7.0) and spiked with defined CAP content. The final CAP concentrations were tested by the DPV technique, and three parallel tests were conducted for every concentration. As listed in Table 2, C-BN/GCE showed satisfactory recoveries and small RSDs (less than 5%), confirming that the proposed method is reliable and applicable.

## 4. Conclusions

In conclusion, a novel and efficient CAP detection method was developed using C-BN nanosheets as sensing materials. The C-BN nanosheets were successfully synthesized via a molten-salt process and well characterized. Modulation of the band structure in defective *h*-BN was observed by carbon doping, which improved the electrochemical performance of C-BN/GCE, such as conductivity and EASA. As a result, C-BN/GCE showed enhanced electrocatalytic activity in the redox of CAP compared to that of *h*-BN. Under the optimized conditions, the proposed method exhibited a low LOD of 0.035 µM and a wide linear range from 0.1 µM to 700 µM in the detection of CAP. Moreover, the good anti-interference ability, high stability, and desired reproducibility of C-BN/GCE were experimentally authenticated. The good recovery results obtained in real sample analyses suggested the practical feasibility of the as-fabricated sensor for CAP detection. Hence, defect-engineered *h*-BN nanomaterials are potential candidates for electrochemical sensing applications.

## Figures and Tables

**Table 1:** Comparison of the analytical performance of C-BN/GCE with previously reported electrochemical methods for CAP detection.

Electrode Material	Method	Linear Range (µM)	LOD (µM)	Refs
rGO@PDA@AuNPs	DPV	0.1–100	0.058	[2]
ZVO/SGN/LGE	DPV	0.005–325.5	0.0024	[44]
Co_3_O_4_@rGO	i-t	0.1–1500	0.1	[48]
Fe_3_O_4_/N-rGO	DPV	1–200	0.03	[49]
Sr-ZnO@rGO	LSV	0.19–2847.3	0.13	[50]
MoN@S-GCN	DPV	0.5–2450	0.0069	[51]
α-Fe_2_O_3_/SPCE	DPV	2.5–50	0.11	[52]
MoS_2_-rGO	DPV	1–55	0.6	[53]
3D-printed CB/PLA	DPV	10–331	0.98	[54]
NiCo_2_O_4_@C	DPV	0.5–320	0.035	[55]
MoS_2_-MWCNTs	DPV	1–35	0.4	[56]
MoS_2_/f-MWCNTs	i-t	0.08–1392	0.015	[57]
Mn_2_O_3_ TNS/SPCE	DPV	0.015–1.28	0.00426	[58]
Eu_2_O_3_/GO	i-t	0.02–800.25	0.00132	[59]
C-BN	DPV	0.1–200, 200–700	0.035	Here

rGO: reduced graphene oxide; PDA: polydopamine; ZVO: ZnV_2_O_8_; SGN: sulfur doped carbon nitride; LGE: laser-induced graphene electrode; Sr-ZnO: Strontium doped zinc oxide; SPCE: screen-printed carbon electrode; S-GCN: sulfur-doped graphitic carbon nitride; CB/PLA: carbon-black integrated polylactic acid; MWCNT: multi-walled carbon nano-tube.

**Table 2:** CAP quantification in real samples using C-BN/GCE sensor (n = 3).

Real Sample	Added (µM)	Founded (µM)	Recovery (%)	RSD (%)
Human serum	0	–	–	–
	20	19.75 ± 0.36	98.8 ± 1.8	3.05
	40	39.68 ± 1.07	99.2 ± 2.7	2.61
	60	61.02 ± 1.79	101.7 ± 2.9	2.93
Eye drops ^1^	0	7.912 ± 0.31	102.3 ± 3.8	3.96
	20	27.65 ± 0.12	99.7 ± 0.4	0.43
	40	47.90 ± 0.82	100.3 ± 1.7	1.70
	60	65.79 ± 1.00	97.1 ± 1.4	1.52

^1^ The stock solution of CAP eye drops is 7.737 µM.

## Data Availability

All the data are presented in the manuscript.

## Supplementary Materials

The following supporting information can be downloaded at https://www.mdpi.com/article/10.3390/bios13010116/s1: Figure S1: EDS profile for C-BN; Figure S2: CVs of (a) bare GCE, (c) *h*-BN/GCE and (e) C-BN/GCE at different scan rate (25–200 mV/s). Plots of the redox peak current (I_p_) versus the square root of the scan rate (υ^1/2^) at (b) bare GCE, (d) *h*-BN/GCE and (f) C-BN/GCE. All tests were conducted in 0.1 M KCl solution containing 1 mM [Fe(CN)_6_]^3−/4−^; Figure S3: The possible electrochemical reaction mechanism of CAP at C-BN/GCE.

## References

[1] Nguyen L.M., Nguyen N.T.T., Nguyen T.T.T., Nguyen T.T., Nguyen D.T.C., Tran T.V. (2022). Occurrence, toxicity and adsorptive removal of the chloramphenicol antibiotic in water: A review. Environ. Chem. Lett..

[2] Zhang L., Yin M., Wei X.X., Sun Y.W., Chen Y., Qi S.Y., Tian X.X., Qiu J.X., Xu D.P. (2022). Synthesis of rGO@PDA@AuNPs for an effective electrochemical chloramphenicol sensor. Diamond Relat. Mater..

[3] Hu X., Qin J.Z., Wang Y.B., Wang J.J., Yang A.J., Yang A.J., Tsang Y.F., Liu B.J. (2022). Synergic degradation Chloramphenicol in photo-electrocatalytic microbial fuel cell over Ni/MXene photocathode. J. Colloid Interface Sci..

[4] Zhang J.Y., Zhao R.X., Cao L.J., Lei Y.S., Liu J., Feng J., Fu W.J., Li X.Y., Li B. (2020). High-efficiency biodegradation of chloramphenicol by enriched bacterial consortia: Kinetics study and bacterial community characterization. J. Hazard. Mater..

[5] Lin J., Zhang K.T., Jiang L.K., Hou J.F., Yu X., Feng M.B., Ye C.S. (2022). Removal of chloramphenicol antibiotics in natural and engineered water systems: Review of reaction mechanisms and product toxicity. Sci. Total Environ..

[6] Hu X., Deng Y., Zhou J.T., Liu B.J., Yang A.J., Jin T., Tsang Y.F. (2020). N-and O self-doped biomass porous carbon cathode in an electro-Fenton system for Chloramphenicol degradation. Sep. Purif. Technol..

[7] Wei L.Y., Jiao F., Wang Z.L., Wu L., Dong D.M., Chen Y.P. (2022). Enzyme-modulated photothermal immunoassay of chloramphenicol residues in milk and egg using a self-calibrated thermal imager. Food Chem..

[8] Vilian A.T.E., Oh S.Y., Rethinasabapathy M., Umapathi R., Hwang S.K., Oh C.W., Park B., Huh Y.S., Han Y.K. (2020). Improve conductivity of flower-like MnWO_4_ on defect engineered graphitic carbon nitride as an efficient electrocatalyst for ultrasensitive sensing of chloramphenicol. J. Hazard. Mater..

[9] Yang J.W., Ji G.Z., Gao Y., Fu W., Irfan M., Mu L., Zhang Y.L., Li A.M. (2020). High-yield and high-performance porous biochar produced from pyrolysis of peanut shell with low-dose ammonium polyphosphate for chloramphenicol adsorption. J. Clean. Prod..

[10] He F., Ma W.C., Zhong D., Yuan Y.X. (2020). Degradation of chloramphenicol by α-FeOOH-activated two different double-oxidant systems with hydroxylamine assistance. Chemosphere.

[11] Sniegocki T., Sikorska M.G., Posyniak A. (2015). Transfer of chloramphenicol from milk to commercial dairy products—Experimental proof. Food Chem..

[12] Elik A., Altunay N. (2022). Chemometric approach for the spectrophotometric determination of chloramphenicol in various food matrices: Using natural deep eutectic solvents. Spectrochim. Acta Part A.

[13] Vuran B., Ulusoy H.I., Sarp G., Yilmaz E., Morgul U., Kabir A., Tartaglia A., Locatelli M., Soylak M. (2021). Determination of chloramphenicol and tetracycline residues in milk samples by means of nanofiber coated magnetic particles prior to high-performance liquid chromatography-diode array detection. Talanta.

[14] Sharma R., Akshath U.S., Bhatt P., Raghavarao K. (2019). Fluorescent aptaswitch for chloramphenicol detection—Quantification enabled by immobilization of aptamer. Sens. Actuator B Chem..

[15] Abnous K., Danesh N.M., Ramezani M., Emrani A.S., Taghdisi S.M. (2016). A novel colorimetric sandwich aptasensor based on an indirect competitive enzyme-free method for ultrasensitive detection of chloramphenicol. Biosen. Bioelectron..

[16] Liu T.S., Xie J., Zhao J.F., Song G.X., Hu Y.M. (2014). Magnetic chitosan nanocomposite used as cleanup material to detect chloramphenicol in milk by GC-MS. Food Anal. Method..

[17] Kim N., Park I.S. (2006). Development of a chemiluminescent immunosensor for chloramphenicol. Anal. Chim. Acta.

[18] Demir E., Silah H. (2020). Development of a new analytical method for determination of veterinary drug oxyclozanide by electrochemical sensor and its application to pharmaceutical formulation. Chemosensors.

[19] Abhishek K.J., Sathish R., Shubha A., Lakshmi B., Deepak K., Naveen C.S., Harish K.N., Ramakrishna S. (2022). A review on nanomaterial-based electrodes for the electrochemical detection of chloramphenicol and furazolidone antibiotics. Anal. Methods.

[20] Dong X.Z., Yan X.H., Li M., Liu H., Li J.W., Wang L., Wang K., Lu X., Wang S.Y., He B.S. (2020). Ultrasensitive detection of chloramphenicol using electrochemical aptamer sensor: A mini review. Electrochem. Commun..

[21] Tyagi D., Wang H.D., Huang W.C., Hu L.P., Tang Y.F., Guo Z.N., Ouyang Z.B., Zhang H. (2020). Recent advances in two-dimensional-material-based sensing technology toward health and environmental monitoring applications. Nanoscale.

[22] Li T., Shang D.W., Gao S.W., Wang B., Kong H., Yang G.Z., Shu W.D., Xu P.L., Wei G. (2022). Two-dimensional Material-based electrochemical sensors/biosensors for food safety and biomolecular detection. Biosensors.

[23] Li M.N., Huang G., Chen X.J., Yin J.N., Zhang P., Yao Y., Shen J., Wu Y.W., Huang J. (2022). Perspectives on environmental applications of hexagonal boron nitride nanomaterials. Nano Today.

[24] Rohaizad N., Mayorga-Martinez C.C., Fojtu M., Latiff N.M., Pumera M. (2021). Two-dimensional materials in biomedical, biosensing and sensing applications. Chem. Soc. Rev..

[25] Ihsanullah I. (2021). Boron nitride-based materials for water purification: Progress and outlook. Chemosphere.

[26] Wang J.M., Zhang L.Z., Wang L.F., Lei W.W., Wu Z.S. (2021). Two-dimensional boron nitride for electronics and energy applications. Energy Environ. Mater..

[27] Lopes J.M.J. (2021). Synthesis of hexagonal boron nitride: From bulk crystals to atomically thin films. Prog. Cryst. Growth Charact. Mater..

[28] Weng Q.H., Wang X.B., Wang X., Bando Y., Golberg D. (2016). Functionalized hexagonal boron nitride nanomaterials: Emerging properties and application. Chem. Soc. Rev..

[29] Angizi S., Khalaj M., Alem S.A.A., Pakdel A., Willander M., Hatamie A., Simchi A. (2020). Review—Towards the two-dimensional hexagonal boron nitride (2D h-BN) electrochemical sensing platforms. J. Electrochem. Soc..

[30] Yin J., Li J.D., Hang Y., Yu J., Tai G.A., Li X.M., Zhang Z.H., Guo W.L. (2016). Boron nitride nanostructures: Fabrication, functionalization and applications. Small.

[31] Gottscholl A., Diez M., Soltamov V., Kasper C., Sperlich A., Kianinia M., Bradac C., Aharnonvich I., Dyakonov V. (2021). Room temperature coherent control spin defects in hexagonal boron nitride. Sci. Adv..

[32] Legesse M., Rashkeev S.N., Saidaoui H., Mellouhi F.E., Ahzi S., Alharbi F.H. (2020). Band gap tuning in aluminum doped two-dimensional hexagonal boron nitride. Mater. Chem. Phys..

[33] Zhang J.J., Sun R., Ruan D.L., Zhang M., Li Y.X., Zhang K., Cheng F.L., Wang Z.C., Wang Z.M. (2020). Point defects in two-dimensional hexagonal boron nitride: Perspective. J. Appl. Phys..

[34] Shen Y.L., Ouyang H.Y., Li W.F., Long Y.M. (2021). Defect-enhanced electrochemical property of h-BN for Pb^2+^ detection. Microchim. Acta.

[35] Ouyang H.Y., Li W.F., Long Y.M. (2021). Carbon-doped h-BN for the enhanced electrochemical determination of dopamine. Electrochim. Acta.

[36] Esrafili M.D., Rad F.A. (2019). Carbon-doped boron nitride nanosheets as highly sensitive materials for detection of toxic NO and NO_2_ gases: A DFT study. Vacuum.

[37] Mudchimo T., Namuangruk S., Kungwan N., Jungsuttiwong S. (2018). Carbon-doped boron nitride nanosheet as a promising metal-free catalyst for NO reduction: DFT mechanistic study. Appl. Catal. A Gen..

[38] Gao M., Adachi M., Lyalin A., Taketsugu T. (2016). Long range functionalization of h-BN monolayer by carbon doping. J. Phys. Chem. C.

[39] Tippo P., Singjai P., Choopun S., Sakulsermsuk S. (2018). Preparation and electrical properties of nanocrystalline BCNO. Mater. Lett..

[40] Lei W.W., Portehault D., Dimova R., Antonietti M. (2011). Boron carbon nitride nanostructures from salt melts: Tunable water-soluble phosphors. J. Am. Chem. Soc..

[41] Huang C.J., Chen C., Zhang M.W., Lin L.H., Ye X.X., Lin S., Antonietti M., Wang X.C. (2015). Carbon-doped BN nanosheets for metal-free photoredox catalysis. Nat. Commun..

[42] Chen S.R., Li P., Xu S.T., Pan X.L., Fu Q., Bao X.H. (2018). Carbon doping of hexagonal boron nitride porous materials toward CO_2_ capture. J. Mater. Chem. A.

[43] Vokhmintsev A., Weinstein I., Zamyatin D. (2019). Electron-phonon interactions in subband excited photoluminescence of hexagonal boron nitride. J. Lumin..

[44] Rajaji U., Govindasamy M., Sha R., Alsjgari R.A., Juang R.S., Liu T.Y. (2022). Surface engineering of 3D spinel Zn_3_V_2_O_8_ wrapped on sulfur doped graphitic nitride composites: Investigation on the dual role of electrocatalyst for simultaneous detection of antibiotic drugs in biological fluids. Compos. Part B.

[45] Kokulnathan T., Sharma T.S.K., Chen S.M., Chen T.W., Dinesh B. (2018). Ex-situ decoration of graphene oxide with palladium nanoparticles for the highly sensitive and selective electrochemical determination of chloramphenicol in food and biological samples. J. Taiwan Inst. Chem. Eng..

[46] Kokulnathan T., Sharma T.S.K., Chen S.M., Yu Y.H. (2018). Synthesis and characterization of Zirconium dioxide anchored carbon nanofiber composite for enhanced electrochemical determination of chloramphenicol in food samples. J. Electrochem. Soc..

[47] Zhao H.M., Chen Y.Q., Tian J.P., Yu H.T., Quan X. (2012). Selectively electrochemical determination of chloramphenicol in aqueous solution using molecularly imprinted polymer-carbon nanotubes-gold nanoparticles modified electrode. J. Electrochem. Soc..

[48] Adav M., Ganesan V., Gupta R., Yadav D.K., Sonkar P.K. (2019). Cobalt oxide nanocrystals anchored on graphene sheets for electrochemical determination of chloramphenicol. Microchem. J..

[49] Qi X., Teng Z.X., Yu J.H., Jia D.L., Zhang Y.F., Pan H.Z. (2023). A simple one-step synthesis of Fe_3_O_4_/N-rGO nanocomposite for sensitive electrochemical detection of chloramphenicol. Mater. Lett..

[50] Selvi S.V., Nataraj N., Chen S.M. (2020). The electro-catalytic activity of nanosphere strontium doped zinc oxide with rGO layers screen-printed carbon electrode for the sensing of chloramphenicol. Microchem. J..

[51] Jaysiva G., Manavalan S., Chen S.M., Veerakumar P., Keerthi M., Tu H.S. (2020). MoN nanorod/sulfur-doped graphitic carbon nitride for electrochemical determination of chloramphenicol. ACS Sustain. Chem. Eng..

[52] Huyen N.N., Dinh N.X., Doan M.Q., Vu N.P., Das R., Le M.T., Thang D., Le A.T. (2022). Unraveling the roles of morphology and steric hindrance on electrochemical analytical performance of α-Fe_2_O_3_ nanostructures-based nanosensors towards chloramphenicol antibiotic in shrimp samples. J. Electrochem. Soc..

[53] Gao S., Yang Z.M., Zhang Y.Q., Zhao L., Xing Y.P., Fei T., Liu S., Zhang T. (2022). The synergistic effects of MoS_2_ and reduced graphene oxide on sensing performance for electrochemical chloramphenicol sensor. FlatChem.

[54] Oliveira M.D., Rocha R.G., Faria L.V.D., Richter E.M., Munoz R.A.A. (2022). Carbon-black integrated polylactic acid electrochemical sensor for chloramphenicol determination in milk and water samples. J. Electrochem. Soc..

[55] Niu X., Bo X.J., Guo L.P. (2021). MOF-derived hollow NiCo_2_O_4_/C composite for simultaneous electrochemical determination of furazolidone and chloramphenicol in milk and honey. Food Chem..

[56] Gao S., Zhang Y.P., Yang Z.M., Fei T., Liu S., Zhang T. (2021). Electrochemical chloramphenicol sensors-based on trace MoS_2_ modified carbon nanomaterials: Insight into carbon supports. J. Alloys Compd..

[57] Govindasamy M., Chen S.M., Mani V., Devasenathipathy R., Umamaheswari R., Santhanaraj K.J., Sathiyan A. (2017). Molybdenum disulfide nanosheets coated multiwalled carbon nanotubes composite for highly sensitive determination of chloramphenicol in food samples milk, honey and powdered milk. J. Colloid Interface Sci..

[58] Rajaji U., Muthumariappan A., Chen S.M., Chen T.W., Tseng T.W., Wang K., Qi D.D., Jiang H.Z. (2019). Facile sonochemical synthesis of porous and hierarchical manganese(III) oxide tiny nanostructures for super sensitive electrocatalytic detection of antibiotic (chloramphenicol) in fresh milk. Ultrason. Sonochem..

[59] Rajaji U., Manavalan S., Chen S.M., Govindasamy M., Maiyalagan T. (2019). Microwave-assisted synthesis of europium(III) oxide decorated reduced graphene oxide nanocomposite for detection of chloramphenicol in food samples. Compos. Part B.

