# Supplementary material for: An Effective Electrochemical Platform for Chloramphenicol Detection Based on Carbon-Doped Boron Nitride Nanosheets
Source: Biosensors (Basel). 2023 Jan 9;13(1):116. doi: 10.3390/bios13010116 (PMC9855874; doi:10.3390/bios13010116)
Supplement: Supplementary file 1 [file biosensors-13-00116-s001.zip › biosensors-2120585-supplementary.pdf]

Supporting information

An Effective Electrochemical Platform for Chloramphenicol Detection Based on Carbon-Doped Boron Nitride Nanosheets

Jingli Yin [1,2], Huiying Ouyang [1,2], Weifeng Li [1] and Yumei Long [1,2,*]

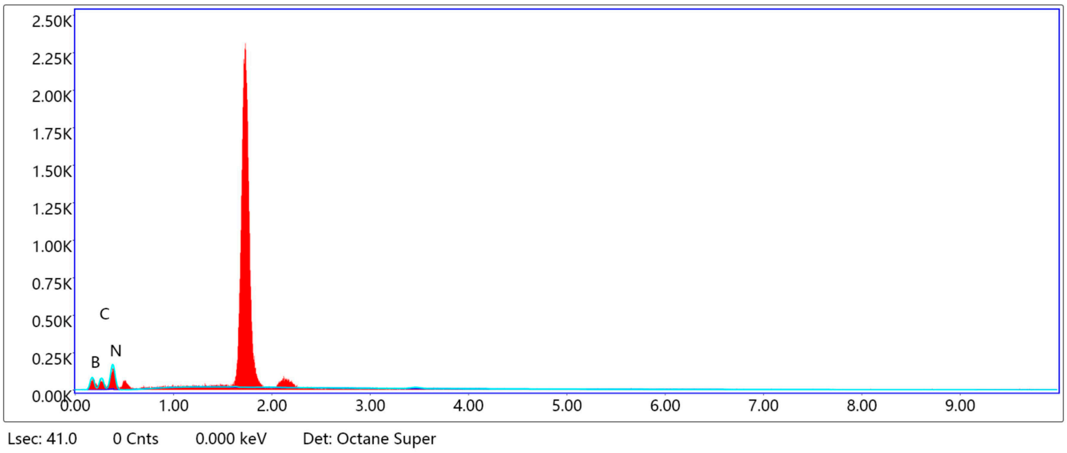

Figure S1. EDS profile for C-BN.

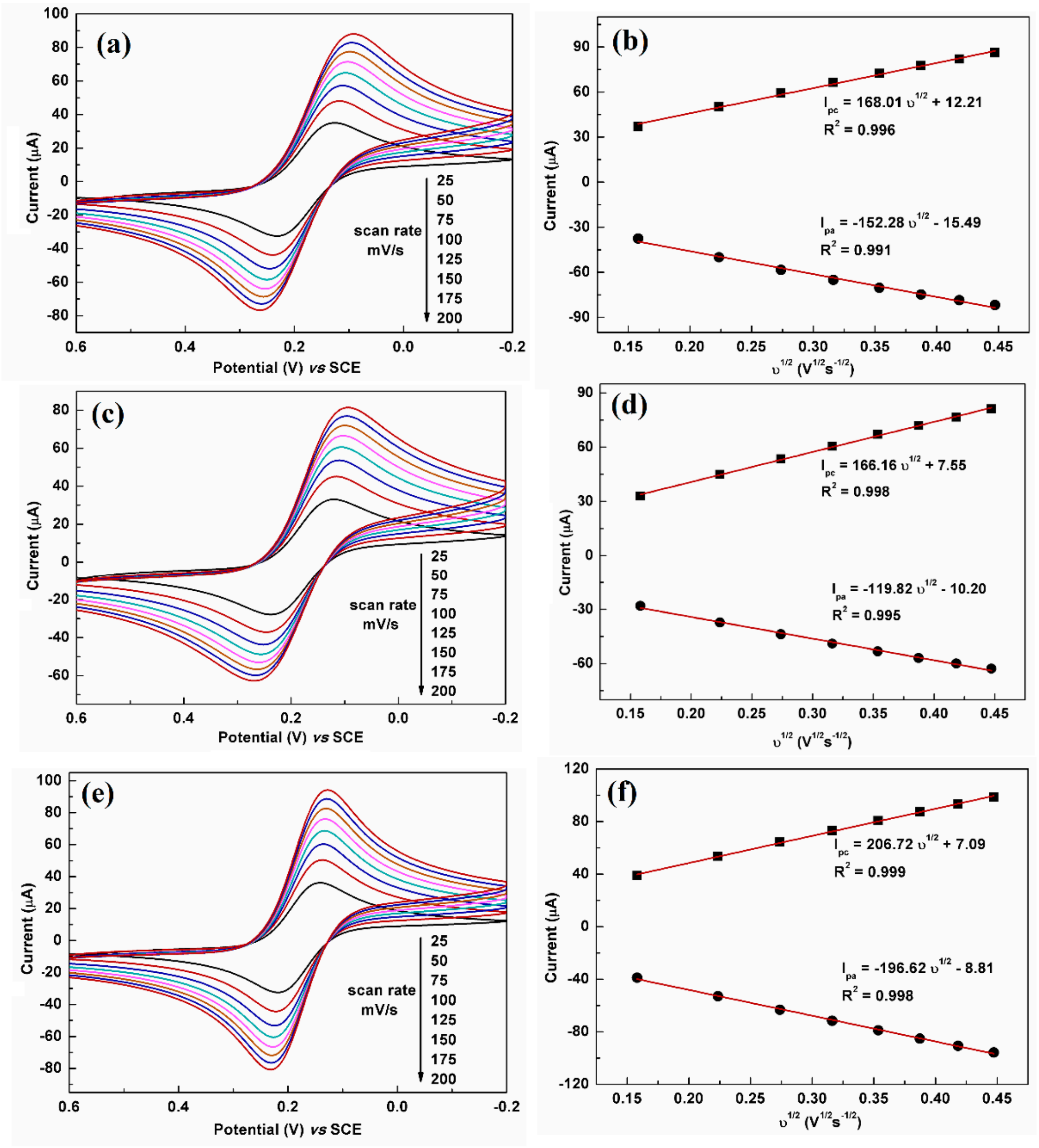

Figure S2. CVs of (a) bare GCE, (c) *h*-BN/GCE and (e) C-BN/GCE at different scan rate (25 – 200 mV/s). Plots of the redox peak current (I_P) versus the square root of the scan rate ($v^{1/2}$) at (b) bare GCE, (d) *h*-BN/GCE and (f) C-BN/GCE. All the tests were conducted in 0.1 M KCl solution containing 1 mM $[Fe(CN)_6]^{3-/4-}$.

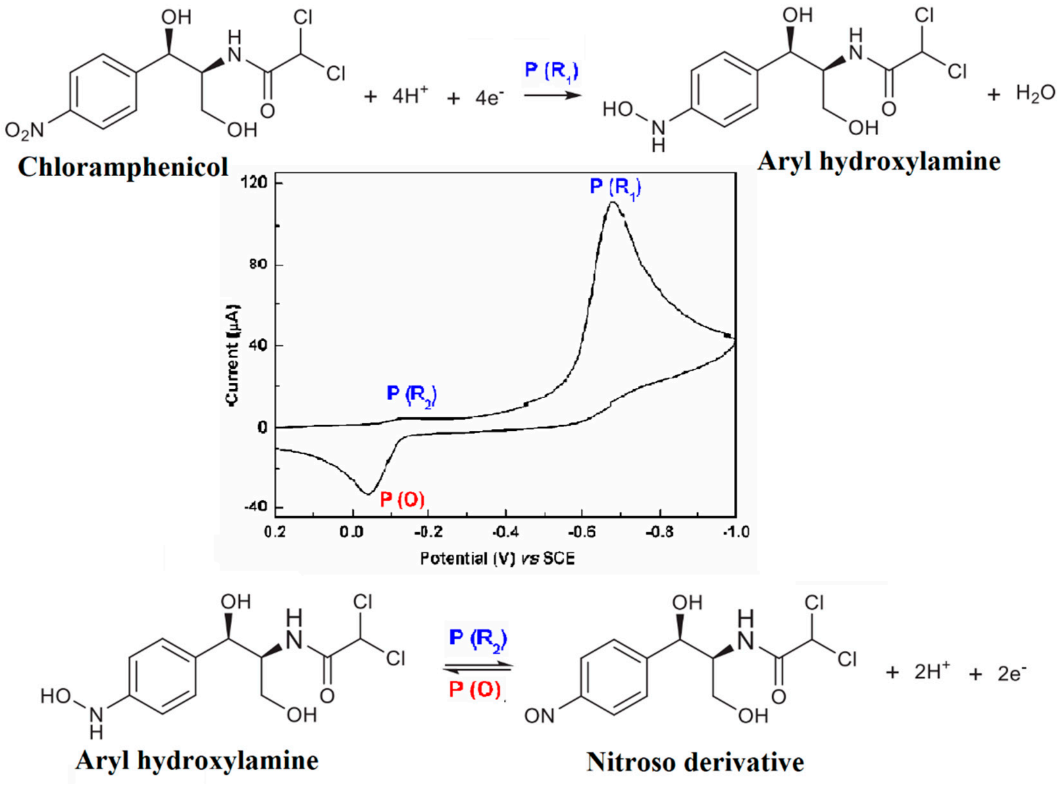

Figure S3. The possible electrochemical reaction mechanism of CAP at C-BN/GCE.
